# Pharmacologic Prophylaxis of Portal Venous System Thrombosis after Splenectomy: A Meta-Analysis

**DOI:** 10.1155/2014/292689

**Published:** 2014-08-27

**Authors:** Xingshun Qi, Ming Bai, Xiaozhong Guo, Daiming Fan

**Affiliations:** ^1^Department of Gastroenterology, General Hospital of Shenyang Military Area, Shenyang 110840, China; ^2^Xijing Hospital of Digestive Diseases, Fourth Military Medical University, Xi'an, Shaanxi 710032, China

## Abstract

Portal venous system thrombosis (PVST) is a life-threatening complication of splenectomy. A meta-analysis was conducted to explore the role of pharmacologic prophylaxis of PVST after splenectomy. Overall, 359 papers were initially identified via the PubMed, EMBASE, and Cochrane Library databases. Eight of them were eligible. The incidence of PVST after splenectomy was significantly lower in patients who received the preventive measures than in those who did not (odds ratio [OR]: 0.33, 95% confidence interval [CI]: 0.22–0.47, *P* < 0.00001). Subgroup analyses demonstrated that the significant difference remained in studies including patients with portal hypertension (*n* = 6), but not in those including patients with hematological diseases (*n* = 2); the significant difference remained in studies using any type of prophylactic drugs (anticoagulants [*n* = 6], thrombolytics [*n* = 1], and prostaglandin E1 [*n* = 1]); the significant difference remained in nonrandomized studies (*n* = 5), but not in randomized studies (*n* = 3). The risk of bleeding was similar between the two groups (OR: 0.65, 95% CI: 0.10–4.04, *P* = 0.64). In conclusion, pharmacologic prophylaxis might decrease the incidence of PVST after splenectomy in patients with portal hypertension and did not increase the risk of bleeding. However, the effect of pharmacologic prophylaxis of PVST in patients with hematological diseases remained questioned.

## 1. Introduction

Portal venous system thrombosis (PVST) is a life-threatening vascular disease characterized by the development of thrombosis within the portal vein, mesenteric vein, and splenic vein [1, 2]. Splenectomy is one of the most common local risk factors of PVST [1, 2]. The incidence of PVST after splenectomy varies from 0% to 50% [3–8]. The heterogeneity is primarily attributable to the different sample sizes, surgical approaches, indications for splenectomy, and diagnostic methods of PVST among studies. The possible mechanism of PVST after splenectomy is the local injury to the vein and its secondary coagulation abnormalities [2]. In addition, the presence of hypercoagulability, weight of spleen, and diameter of splenic vein are considered as the major predisposing factors of PVST after splenectomy.

Pharmacologic prophylaxis may be helpful to decrease the incidence of PVST after splenectomy, thereby reducing its related morbidity and mortality. However, the risk of bleeding secondary to the use of anticoagulants or thrombolytics immediately after splenectomy should be cautioned [9–11], especially in cirrhotic patients with portal hypertension and hypersplenism. Until now, no study has systematically evaluated the efficacy and safety of the pharmacologically preventive measures for the development of PVST after splenectomy. Herein, we conducted a systematic review and meta-analysis to explore this issue.

## 2. Methods

This work was performed according to the PRISMA statement for reporting systematic reviews and meta-analyses of studies that evaluate health care interventions [12].

### 2.1. Search Strategy and Selection Criteria

We performed the literature search via the PubMed, EMBASE, and Cochrane Library databases (from the database inception to October 30, 2013). Additional relevant literature was identified by hand-searching the reference lists of identified literature. Search items were listed as follows: (“anticoagulation” [All Fields]* or* “anticoagulant” [All Fields]* or *“warfarin” [All Fields]* or* “heparin” [All Fields]* or* “low molecular weight heparin” [All Fields]* or* “LMWH”* or* “enoxaparin” [All Fields]* or* “thrombolysis” [All Fields]* or* “thrombolytic” [All Fields]* or* “lytic” [All Fields]* or* “urokinase” [All Fields]* or* “streptokinase” [All Fields]* or* “antithrombotic” [All Fields])* AND* (“splenectomy” [All Fields])* AND* (“splenic vein” [All Fields]* or* “splenic venous” [All Fields]* or* “portal vein” [All Fields]* or* “portal venous” [All Fields]* or* “mesenteric vein” [All Fields]* or* “mesenteric venous” [All Fields])* AND* (“thrombosis” [All Fields]* or* “thrombus” [All Fields]* or* “thrombotic” [All Fields]* or* “occluded” [All Fields]* or* “occlusive” [All Fields]* or* “occlusion” [All Fields]* or* “obstructed” [All Fields]* or* “obstructive” [All Fields]* or* “obstruction” [All Fields]).

We selected the papers according to the following eligibility criteria. (1) Both randomized controlled trials and nonrandomized studies were considered, if the incidence of PVST after splenectomy was compared between patients who received the preventive measures and those who did not. (2) Narrative reviews, systematic reviews, meta-analyses, comments, editorials, animal studies, and case reports were excluded. (3) Studies unrelated to the prevention of PVST after splenectomy were excluded. (4) Studies without any detailed information regarding the prevention of PVST after splenectomy were excluded. (5) Studies with all included patients receiving the prevention of PVST after splenectomy were excluded. (6) There was no publication date, publication language, or publication status restriction.

### 2.2. Data Extraction

We extracted the following data into Excel tables, including the author, journal, publication year, region where a study was conducted, period of enrollment, study design, study population, type of surgery, information regarding the prevention of PVST after splenectomy, type and number of participants, inclusion and exclusion criteria, demographic data (age and sex), type of underlying diseases, liver function (the data was collected in patients with liver diseases, if available), incidence, location, degree of PVST after splenectomy, and incidence of bleeding after the implementation of pharmacologic prophylaxis. We also contacted the authors about the data that were not shown in their papers.

### 2.3. Assessment of Study Quality

We used the Cochrane Collaboration's tool version 5.1.0 and Newcastle-Ottawa scale to evaluate the quality of randomized and nonrandomized studies, respectively.

The Cochrane Collaboration's tool for assessing the risk of bias includes 6 domains, such as selection bias (i.e., random sequence generation and allocation concealment), performance bias (i.e., blinding of participants and personnel), detection bias (i.e., blinding of outcome assessment), attrition bias (i.e., incomplete outcome data), reporting bias (i.e., selective reporting), and other biases (i.e., other sources of bias). For each entry, a study can be judged as low, high, or unclear risk of bias.

The Newcastle-Ottawa scale includes 3 categories, such as selection, comparability, and outcome [13]. For each item within the selection and exposure categories, a study can be awarded a maximum of one star; for the comparability category, a study can be awarded a maximum of two stars.

Study quality was independently assessed by two authors. When there were any disagreements, a consensus was reached by discussion with each other.

### 2.4. Statistical Analysis

The number of PVST and bleeding events and total observed participants in two groups were extracted from each study. Then, odds ratio (OR) with 95% confidence interval (CI) was calculated. Finally, the OR of each study was pooled, using either fixed-effects (Mantel-Haenszel method) [14] or random-effects model (DerSimonian-Laird method) [15]. When the heterogeneity among studies was not significant, we used the fixed-effects model to calculate the pooled data. Otherwise, we used the random-effects model. Additionally, subgroup analyses were performed to identify the efficacy of pharmacologic prophylaxis according to the type of study population (hematological diseases or portal hypertension), type of drugs used for the prevention of PVST (anticoagulants, thrombolytics, or others), and study design (randomized or nonrandomized studies). Heterogeneity among studies was assessed by using the *I* ^2^ statistic (*I* ^2^ > 50% was considered as having substantial heterogeneity) and the Chi-square test (*P* < 0.10 was considered to represent significant statistical heterogeneity) [16]. Sensitivity analyses were performed by sequential omission of every individual study to explore the cause of heterogeneity among studies. Funnel plots were used to assess the publication bias. All analyses were conducted using the statistical package Review Manager version 5.1 (Copenhagen, The Nordic Cochrane Center, The Cochrane Collaboration, 2011).

## 3. Results

**Figure 1:**
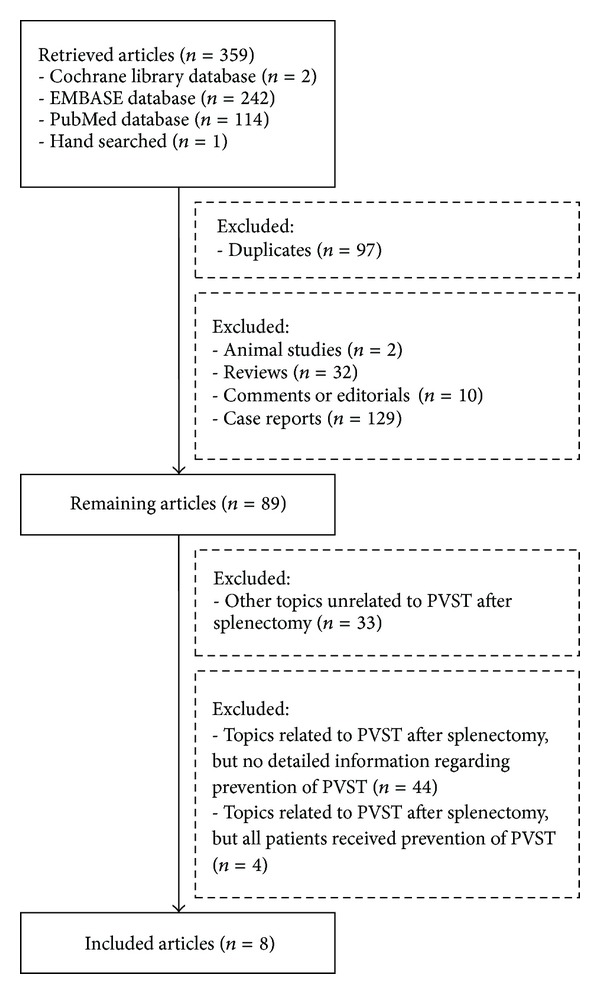
Flowchart of study inclusion.

Overall, 359 papers were initially identified. After exclusion, 8 papers were included in our meta-analysis (Figure 1) [17–24]. Notably, a randomized controlled trial by Wang et al. was terminated due to the absence of funding support [23]. According to the preplanned study protocol, the study would require 214 participants (107 per arm). However, only 35 patients were finally enrolled. Additionally, in a retrospective study by Lai et al., regular and irregular anticoagulation were considered as the exposed and nonexposed group, respectively [19]. In detail, regular anticoagulation referred to subcutaneous injection of LMWH followed by oral warfarin; by comparison, irregular anticoagulation referred to aspirin or warfarin monotherapy for an undesignated time period without LMWH. Given the significant difference of drugs used between the two groups, this study was considered eligible to evaluate the effect of anticoagulation for the prevention of PVST after splenectomy.

These included studies were published in full-texts (*n* = 7) or abstract (*n* = 1) between 2000 and 2013 (Table 1). Of them, 3 were randomized trials [20, 23, 24], 2 were prospective cohort studies [17, 18], and 3 were retrospective cohort studies [19, 21, 22]. These studies were conducted by the investigators from Canada (*n* = 1), China (*n* = 4), Japan (*n* = 2), and Sweden (*n* = 1). In 6 Asian studies from China and Japan, the patients undergoing splenectomy were diagnosed with liver cirrhosis, portal hypertension, and/or hypersplenism [17–21, 24]. In 2 Western studies from Canada and Sweden, the patients undergoing splenectomy were diagnosed with hematological diseases [22, 23]. The information regarding the eligibility criteria of patients was summarized in Supplementary Table 1 available online at http://dx.doi.org/10.1155/2014/292689. The detailed information regarding the prevention of PVST after splenectomy was summarized in Supplementary Table 2. Among them, anticoagulants were employed in 6 studies, thrombolytics in 1 study, and prostaglandin E1 in 1 study.

Patient characteristics of these included studies were summarized in Supplementary Table 3. Although we contacted Ma and Pan for the data regarding sex, age, and liver function, none replied. Among these studies, 2 studies did not provide the data regarding the sex and age [20, 21], and 1 study including patients with portal hypertension did not provide the data regarding liver function [21].

### 3.1. Study Quality

#### 3.1.1. Randomized Studies

For the selection bias, 1 and 2 studies were at a low and unclear risk, respectively (Supplementary Table 4). For the performance, 2 studies were at an unclear risk, and another study was at a high risk because it was an open-label study. For the detection bias, 3 studies were at a low risk. For the attrition bias, 2 studies were at an unclear risk, and another study was at a high risk because it was an underpowered study. For the reporting and other biases, all of the 3 studies had an unclear risk.

#### 3.1.2. Nonrandomized Studies

Two and 3 studies were awarded <5 and ≧5 stars, respectively (Supplementary Table 5).

### 3.2. Incidence of PVST

**Figure 2:**
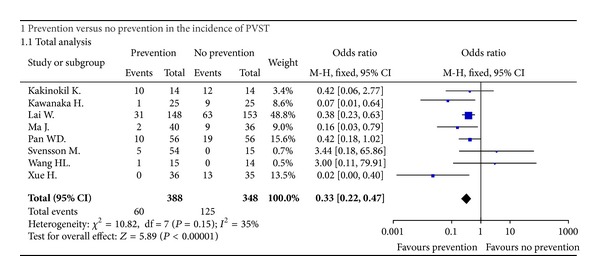
Forest plots showing the results of meta-analysis comparing the incidence of portal venous system thrombosis after splenectomy between patients who received the preventive measures and those who did not.

**Figure 3:**
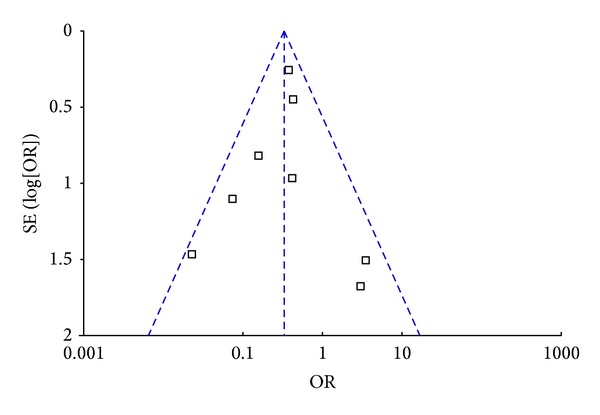
Funnel plot to explore the publication bias in the meta-analyses comparing the incidence of portal venous system thrombosis after splenectomy between patients who received the preventive measures and those who did not.

All of the 8 included studies provided the data regarding the incidence of PVST after splenectomy between patients who received the preventive measures and those who did not. Heterogeneity among studies was not significant (*I* ^2^ = 35%, *P* = 0.15). Using a fixed-effect model, the pooled OR was 0.33 (95% CI: 0.22–0.47, *P* < 0.00001) (Figure 2), suggesting a significantly lower incidence of PVST after splenectomy in patients who received the preventive measures. Funnel plots demonstrated all studies laid within 95%CI, suggesting no proof of publication bias (Figure 3).

#### 3.2.1. Subgroup Analysis according to the Type of Study Population

In the subgroup analysis of 2 studies including the patients with hematological diseases, only anticoagulant drugs were used for the prevention of PVST. The heterogeneity among studies was not significant (*I* ^2^ = 0%, *P* = 0.95). Using a fixed-effect model, the pooled OR was 3.27 (95% CI: 0.36–29.57, *P* = 0.29) (Supplementary Figure 1), suggesting that the use of anticoagulants might not significantly decrease the incidence of PVST after splenectomy in patients with hematological diseases.

In the subgroup analysis of 6 studies including the patients with portal hypertension, 3 different types of drugs were used for the prevention of PVST. The heterogeneity among studies was not significant (*I* ^2^ = 28%, *P* = 0.22). Using a fixed-effect model, the pooled OR was 0.29 (95% CI: 0.20–0.43, *P* < 0.00001) (Supplementary Figure 1), suggesting that the implementation of pharmacologic prophylaxis could significantly reduce the incidence of PVST after splenectomy in patients with portal hypertension.

#### 3.2.2. Subgroup Analysis according to the Type of Drugs Used for the Prevention of PVST

In the subgroup analysis of 6 studies using anticoagulants, the heterogeneity was not significant (*I* ^2^ = 15%, *P* = 0.32). Using a fixed-effect model, the pooled OR was 0.40 (95% CI: 0.27–0.59, *P* < 0.00001) (Supplementary Figure 2), suggesting that the use of anticoagulants could significantly decrease the incidence of PVST after splenectomy.

In the subgroup analysis of 1 study using thrombolytics, the pooled OR was 0.02 (95% CI: 0.001–0.40, *P* = 0.010) (Supplementary Figure 2), suggesting that the use of thrombolytics could significantly decrease the incidence of PVST after splenectomy.

In the subgroup analysis of 1 study using prostaglandin E1, the pooled OR was 0.16 (95% CI: 0.03–0.79, *P* = 0.02) (Supplementary Figure 2), suggesting that the use of prostaglandin E1 could significantly decrease the incidence of PVST after splenectomy.

#### 3.2.3. Subgroup Analysis according to the Study Design

In the subgroup analysis of 3 randomized studies, the heterogeneity was significant (*I* ^2^ = 59%, *P* = 0.09). Using a random-effect model, the pooled OR was 0.19 (95% CI: 0.02–1.89, *P* = 0.16) (Supplementary Figure 3), suggesting a similar incidence of PVST after splenectomy between the two groups.

In the subgroup analysis of 5 nonrandomized studies, the heterogeneity was not significant (*I* ^2^ = 9%, *P* = 0.35). However, given the consistency of statistical methods between the two subgroups analyses, we still used a random-effect model to calculate a more conservative result. The pooled OR was 0.38 (95% CI: 0.24–0.63, *P* = 0.00001) (Supplementary Figure 3), suggesting a significantly lower incidence of PVST after splenectomy in patients who received the preventive measures.

### 3.3. Risk of Bleeding

**Figure 4:**
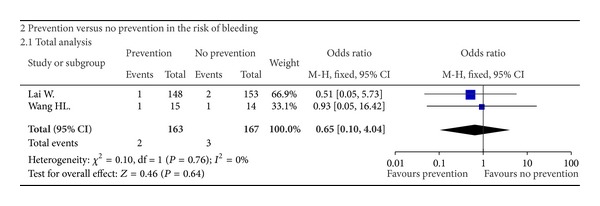
Forest plots showing the results of meta-analysis comparing the risk of bleeding between patients who received the preventive measures and those who did not.

Only 2 studies provided the data regarding the incidence of bleeding between patients who received the preventive measures and those who did not. Heterogeneity among studies was not significant (*I* ^2^ = 0%, *P* = 0.67). Using a fixed-effect model, the pooled OR was 1.51 (95% CI: 0.24–9.27, *P* = 0.66) (Figure 4), suggesting a similar incidence of bleeding between the two groups. Funnel plots were not performed due to a very small number of studies included.

## 4. Discussion

Our systematic review and meta-analysis primarily aimed to compare the incidence of PVST and risk of bleeding between patients who received the preventive measures for the development of PVST after splenectomy and those who did not. A major finding of our study was that the drugs, including anticoagulants, thrombolytics, and prostaglandin E1, could significantly decrease the incidence of PVST after splenectomy. However, according to the results of subgroup analyses, we found that the efficacy of pharmacologic prophylaxis of PVST after splenectomy remained significant in patients with portal hypertension and hypersplenism but might disappear in those with hematological diseases. This unexpected finding potentially questioned the necessity of pharmacologic prophylaxis of PVST after splenectomy in patients with hematological diseases. But it should be noted that only 2 studies with a small sample size explored this issue in patients with hematological diseases. Additionally, the number of PVST events was very small in the 2 studies, which potentially hampered from achieving a statistical significance. As mentioned by Wang et al. [23], a low incidence of PVST in their study might be contributed by a relatively low proportion of participants with myeloproliferative disease, lymphoproliferative disorders, and hereditary hemolytic anemia. In addition, our meta-analysis showed that the efficacy of preventive measures was significant in nonrandomized studies, but not in randomized studies. However, as we closely looked at the results of the subgroup analysis of 3 randomized studies, there was a trend towards favoring the use of pharmacologic prophylaxis of PVST after splenectomy. In detail, 2 of 3 randomized studies have shown a significant benefit of the preventive measures after splenectomy.

Theoretically, the patients with portal hypertension and hypersplenism, especially those with a lower platelets count and undergoing abdominal surgery, have a tendency of bleeding. Under this circumstance, the use of anticoagulation or thrombolysis after splenectomy in such patients often represents a clinical challenge. By contrast, our meta-analysis demonstrated that the risk of bleeding might not be increased after the implementation of preventive measures. Notably, this finding should be interpreted with caution due to the limited data from only 2 studies.

Several important risk factors of predicting the development of PVST after splenectomy have been increasingly recognized. They mainly include a greater spleen weight, a wider portal or splenic vein diameter, a higher D-dimer and P-selectin level, use of laparoscopic technique, and presence of hemolytic anemia or myeloproliferative disorders [3–8]. Accordingly, the early implementation of pharmacologic prophylaxis of PVST after splenectomy may be more reasonable and cost-effective in patients with the abovementioned risk factors. However, in our systematic review and meta-analysis, no relevant data regarding the efficacy of preventive measures in these “high-risk” patients could be extracted from these included studies. Thus, further studies should focus on such patients.

Compared with surgical splenectomy, partial splenic artery embolization is less invasive without general anesthesia [25]. It has been gradually considered as an alternative to surgical splenectomy in patients with portal hypertension and hypersplenism [25, 26]. Recent studies have shown that the efficacy of partial splenic artery embolization is comparable to that of splenectomy for the treatment of hypersplenism secondary to chronic liver disease [27]. Notably, the presence of PVST after partial splenic artery embolization remains a major postoperative complication. Whether or not the benefit of preventive measures for the development of PVST in patients undergoing splenectomy could be extrapolated to those undergoing partial splenic embolization should be further confirmed.

The major limitation of our study was the small number of studies included in our meta-analysis, which greatly limited the reliability of the findings from the meta-analyses regarding the risk of bleeding after the implementation of pharmacologic prophylaxis of PVST and the efficacy of preventive measures after splenectomy in patients with hematological diseases. Indeed, as known, the inclusion of nonrandomized studies would result in the selection and reporting bias. However, this behavior was primarily attributed to such a small number of included studies. In addition, the quality of these included studies was relatively low. Of the 3 randomized studies, 2 did not describe any detailed methods of randomization and sample size calculation and 1 was aborted in which the sample size needed for an adequate power was not met due to the low enrollment. Given these study limitations, the findings must be interpreted with caution.

## 5. Conclusion

The implementation of pharmacologic prophylaxis could significantly decrease the incidence of PVST after splenectomy in patients with portal hypertension and hypersplenism and might not increase the risk of bleeding. However, owing to the limited evidence from 2 studies, the efficacy of anticoagulation for the prevention of PVST after splenectomy in patients with hematological diseases remained questioned. Thus, further well-designed randomized studies in different study population should be warranted to provide a confirmative recommendation.

## Supplementary Material

Supplementary Figure 1: Forest plots showing the results of subgroup meta-analyses according to the type of study population comparing the incidence of portal venous system thrombosis after splenectomy between patients who received the preventive measures and those who did not.
Supplementary Figure 2. Forest plots showing the results of subgroup meta-analyses according to the type of drugs comparing the incidence of portal venous system thrombosis after splenectomy between patients who received the preventive measures and those who did not.
Supplementary Figure 3. Forest plots showing the results of subgroup meta-analyses according to the study design comparing the incidence of portal venous system thrombosis after splenectomy between patients who received the preventive measures and those who did not.

## Figures and Tables

**Table 1:** Characteristics of included studies.

First author and Journal (year)	Regions	Enrollment period	Study design	Study population	Surgery	Number of patients
Kakinoki Surg Today (2013) Full-text [17]	Kagawa, Japan	February 2008–April 2011	Prospective	Consecutive patients with LC and hypersplenism. Detailed indications were as follows: a bleeding tendency (*n* = 3), induction of interferon therapy (*n* = 8), difficulties in therapies for HCC due to thrombocytopenia (*n* = 12), and esophagogastric varices (*n* = 5).	Hand-assisted laparoscopic splenectomy	28

Kawanaka Ann Surg (2010) Full-text [18]	Fukuoka, Japan	January 2005–December 2005 (1st period); January 2006–July 2006 (2nd period)	2-period, prospective cohort study	Consecutive patients with LC and hypersplenism. Detailed indications were as follows: bleeding tendency due to thrombocytopenia (*n* = 7), difficulties in the induction or continuation of pegylated interferon therapy plus ribavirin due to thrombocytopenia (*n* = 29), difficulties with therapies for HCC due to thrombocytopenia (*n* = 7), and endoscopic treatment-resistant esophagogastric varices (*n* = 7).	Laparoscopic splenectomy	50

Lai World J Gastroenterol (2012) Full-text [19]	Beijing, China	April 2004–July 2010	Retrospective	Patients with PH, splenomegaly, and hypersplenism. Detailed indications were as follows: hypersplenism and recurrent upper GI bleeding (236 patients had a history of upper GI bleeding).	Splenectomy with gastroesophageal devascularization	301

Ma Zhonghua Yi Xue Za Zhi (2008) Full-text [20]	Xi'an, China	July 2004–August 2005	RCT	Patients with LC and PH.	Splenectomy and pericardial devascularization	76

Pan J Gastroenterol Hepatol (2011) Abstract [21]	Guangzhou, China	March 1999–June 2005	Retrospective	Patients with PH in LC from hepatitis.	Simple splenectomy, splenectomy and EVL, splenectomy and porta-azygous devascularization	112

Svensson Eur J Haematol (2006) Full-text [22]	Stockholm, Sweden	January 1999–December 2003	Retrospective	Adult patients, age ≧20 yr, who underwent splenectomy for haematological disorders.	Laparoscopic splenectomy (*n* = 39); open splenectomy (*n* = 30)	69

Wang Can J Surg (2011) Full-text [23]	Alberta, Canada	November 2006–November 2008	2-centre, phase II, prospective, open-label, parallel-assignment RCT	No detailed information regarding eligible patients. Patients requiring splenectomy due to various causes (hematological diseases).	Laparoscopic splenectomy	29∗

Xue Zhonghua Wai Ke Za Zhi (2000) Full-text [24]	Zhengzhou, China	June 1995–June 1999	RCT	HBV-related LC patients with PH, upper GI bleeding, splenomegaly, and hypersplenism.	Splenectomy with porta-azygous devascularization	71

Notes: ∗35 participants were enrolled in this RCT. But 6 participants were excluded, because 4 withdrew from the study, 1 required conversion to an open approach, and 1 died at 3 postoperative months from myocardial infarction that was unrelated to the procedure or study medication.

Abbreviations: EVL: endoscopic variceal ligation; GI: gastrointestinal; HBV: hepatitis B virus; LC: liver cirrhosis; PH: portal hypertension; and RCT: randomized controlled trial.
