# Supplementary material for: Pharmacologic Prophylaxis of Portal Venous System Thrombosis after Splenectomy: A Meta-Analysis
Source: Gastroenterol Res Pract. 2014 Aug 27;2014:292689. doi: 10.1155/2014/292689 (PMC4163290; doi:10.1155/2014/292689)

Supplementary Table 1. Eligibility criteria of patients.

First author. Journal. (Year)	Eligibility criteria of patients
Kakinokil K. Surg Today (2013) Full-text	Consecutive patients with cirrhosis and hypersplenism undergoing hand-assisted laparoscopic splenectomy in Kagawa University Hospital from February 2008 through April 2010. All the patients were referred from the Department of Gastroenterology in the same hospital with the data related to liver functional reserve estimation, etiology of liver cirrhosis, accompanying hepatocellular carcinoma and esophageal varix.
Kawanaka H. Ann Surg (2010) Full-text	Consecutive patients with cirrhosis and hypersplenism who underwent laparoscopic splenectomy in Kyushu University in the period from January 2005 to July 2006.
Lai W. World J Gastroenterol. (2012) Full-text	*Inclusion criteria:* patients with liver cirrhosis due to any causes; liver function grade: Child-Pugh A-B; splenomegaly and hypersplenism; severe esophageal varicose confirmed by gastroscopy; and previous histories of recurrent upper gastrointestinal bleeding. All patients signed the informed consent.
	Exclusion criteria: patients who did not fulfll the inclusion criteria and could not tolerate surgical treatment were excluded.
Ma J. Zhonghua Yi Xue Za Zhi (2008) Full-text	Patients with portal hypertension undergoing splenectomy and pericardial devascularization.
Pan WD. J Gastroenterol Hepatol (2011) Abstract	Patients with portal hypertension in liver cirrhosis from hepatitis who had simple splenectomy, splenectomy and EVL, splenectomy and portaazygous devascularization were reviewed from March 1999 to June 2005.
Svensson M. Eur J Haematol (2006) Full-text	Adult patients, age≧20 yr, underwent splenectomy for haematological disorders in the Department of Surgery at Huddinge University Hospital. The patients were selected retrospectively by examining the records in the Department of surgery databank for the splenectomy operation code and the pathological anatomy diagnosis register in the Department of Pathology for the spleen preparation code.
Wang HL. Can J Surg (2011) Full-text	*Inclusion criteria:* 1. undergo laparoscopic splenectomy at The University of Alberta or Grey Nun's Community Hospitals; 2. sign a statement of informed consent; 3. willing to undergo daily subcutaneous injections of Lovenox®.
	Exclusion Criteria: 1. pregnant or nursing; 2. unable or unwilling to provide informed consent; 3. bleeding diathesis or currently on anticoagulation therapy (i.e. coumadin, heparin, low molecular weight heparins); 4. hemorrhagic cerebral vascular accident; 5. severe uncontrolled hypertension; 6. diabetic or hemorrhagic retinopathy; 7. contradictions to anticoagulation (i.e. active gastrointestinal bleed, gastric or duodenal ulcer, sustained platelet count < 50 x10^3/uL, splenectomy due to trauma or history of heparin induced thrombocytopenia); 8. conversion to open splenectomy; 9. allergy to Lovenox®, heparin, or other low molecular weight heparins; 10. bacterial endocarditis.
Xue H. Zhonghua Wai Ke Za Zhi. (2000) Full-text	All patients had hepatitis B virus related liver cirrhosis, portal hypertension, splenomegaly, hypersplenism, esophageal varices, and a history of upper gastrointestinal bleeding. No thrombosis within portal venous system before surgery was observed by color Doppler ultrasound.

Supplementary Table 2. Detailed information regarding the prevention of portal venous system thrombosis after splenectomy.

First author. Journal. (Year)	Criteria of prevention	Timing of prevention	Methods of prevention
Kakinokil K. Surg Today (2013) Full-text	Prophylactic anticoagulation therapy was given at the discretion of each surgeon.	Postoperative day 1 and 2.	Intravenous heparin (10000 U/d) followed by warfarin.
Kawanaka H. Ann Surg (2010) Full-text	During the first period, patients received no prophylactic therapy. During the second period, patients received prophylactic therapy.	Postoperative day 1, 2, and 3.	Intravenous infusion of 1500 units of antithrombin III concentrates.
Lai W. World J Gastroenterol. (2012) Full-text	Irregular anticoagulation was given due to poor blood clotting after surgery and perioperative abdominal or GI bleeding. Regular anticoagulation was given to the patients with nearly normal blood clotting before and after surgery.	24 hours after surgery.	Subcutaneous injection of LMWH routinely, 0.3 ml per 12 hours for 5 days and then maintained by oral therapy with warfarin for one month to keep the target PT/INR at a level between 1.25 and 1.5.
Ma J. Zhonghua Yi Xue Za Zhi (2008) Full-text	Anticoagulation was given according to the randomization. But no detailed information regarding the randomization methods was provided.	Postoperative day 3.	Compared with the control group, the treatment group was added with alprostadil (Lipo Prostaglandin E_1) 20μg once a day for 2 weeks.
Pan WD. J Gastroenterol Hepatol (2011) Abstract	Information was not provided.	Anticoagulation was given as PLT is more than 300×10^9/L (n=33) or in the earlier time (n=23) (no detailed information regarding the date when anticoagulation was initiated).	Anticoagulation was given, but no detailed information regarding the type and dosage of anticoagulants was provided.
Svensson M. Eur J Haematol (2006) Full-text	Patients undergoing LS because of ITP were not routinely given thromboembolic prophylaxis. All other patients splenectomised with LS or OS were given anticoagulants.	Pre- and post-operation.	Thromboembolic prophylaxis was subcutaneous injection of dalteparin 5000 IU once or 2500 IU twice daily until the day of discharge or longer, in median 7 days (range 1–30).
Wang HL. Can J Surg (2011) Full-text	Anticoagulation was given according to the randomization. Patients were randomly assigned to the two groups using a random number table and sealed envelope containing postoperative instructions.	Post-operation.	Patients received 40 mg of enoxaparin subcutaneously once a day for 21 days; and patients with severe renal impairment received an adjusted dose of enoxaparin (a 30-mg subcutaneous dose daily).
Xue H. Zhonghua Wai Ke Za Zhi. (2000) Full-text	Anticoagulation was given according to the randomization. But no detailed information regarding the randomization methods was provided.	Post-operation.	Urokinase 20000 U was infused via a tube inserted into the splenic vein branch every day for 14 days.

Abbreviations: GI, gastrointestinal; INR, international normalized ratio; ITP, idiopathic thrombocytopenic purpura; LMWH, low molecular weight heparin; LS, laparoscopic splenectomy; OS, open splenectomy; PLT, platelets count; PT, prothrombin.

Supplementary Table 3. Patient characteristics of included studies.

First author. Journal. (Year)	Groups	No. Pts	Sex (M/F)	Age (yrs)	Type of diseases	Liver function
Kakinokil K. Surg Today (2013) Full-text	No prevention	14	17/11	Mean±SD (range): 63±8.2 (45-76)	LC with hypersplenism	Child-Pugh class A/B/C: 11/15/2
	Prevention	14				
Kawanaka H. Ann Surg (2010) Full-text	No prevention	25	16/9	Median (range): 56 (41-71)	HBV-LC (n=3); HCV-LC (n=20); alcoholic LC (n=2)	Child-Pugh score: median (range): 7 (5-10)
	Prevention	25	10/15	Median (range): 61 (45-76)	HBV-LC (n=1); HCV-LC (n=21); alcoholic LC (n=1); other (n=2)	Child-Pugh score: median (range): 6.8 (5-10)
Lai W. World J Gastroenterol. (2012) Full-text	Irregular anticoagulation	153	103/50	Mean±SD: 46.14±10.39	HBV-LC (n=128); HCV-LC (n=11); alcoholic LC (n=11); IPH (n=1); biliary LC (n=2)	Child-Pugh class A/B: 121/32
	Regular anticoagulation	148	99/49	Mean±SD: 46.47±9.58	HBV-LC (n=126); HCV-LC (n=8); alcoholic LC (n=11); IPH (n=2); biliary LC (n=1)	Child-Pugh class A/B: 125/23
Ma J. Zhonghua Yi Xue Za Zhi (2008) Full-text	No prevention	36	NA	NA	LC and PH	TBIL: 20.7±4.8 μmol/L; ALT: 32±10 U/L; PT: 14.1±1.8 s
	Prevention	40	NA	NA	LC and PH	TBIL: 21.4±4.2 μmol/L; ALT: 35±8 U/L; PT: 13.6±1.5 s
Pan WD. J Gastroenterol Hepatol (2011) Abstract	No prevention	56	NA	NA	Hepatitis-LC	NA
	Prevention	56	NA	NA	Hepatitis-LC	NA
Svensson M. Eur J Haematol (2006) Full-text	No prevention	15	37/32	Median (range): 56 (20-80)	Idiopathic thrombocytopenic purpura (n=14); haematological malignancy (n=1)	NA
	Prevention	54			Idiopathic thrombocytopenic purpura (n=11); autoimmune haemolytic anaemia (n=5); haematological malignancy (n=36); thalassaemia (n=1); chronic neutropenia (n=1)	NA
Wang HL. Can J Surg (2011) Full-text	No prevention	14	5/9	Median (range): 46 (18-74)	Immune thrombocytopenic purpura (n=10); thrombotic thrombocytopenic purpura (n=2); chronic lymphocytic leukemia (n=1); lymphoma (n=1)	NA
	Prevention	15	11/4	Median (range): 59 (17-72)	Immune thrombocytopenic purpura (n=5); thrombotic thrombocytopenic purpura (n=1); chronic lymphocytic leukemia (n=2); lymphoma (n=2); hereditary spherocytosis (n=1); other (n=4)	NA

Xue H. Zhonghua Wai Ke Za Zhi. (2000) Full-text	No prevention	35	26/9	Mean (range): 41.2 (21-60)	HBV-LC (n=35)	Child-Pugh class A/B/C: 5/22/8
	Prevention	36	26/10	Mean (range): 40.8 (20-59)	HBV-LC (n=36)	Child-Pugh class A/B/C: 6/21/9

Abbreviations: ALT, alanine aminotransferase; F, female; HBV, hepatitis B virus; HCV, hepatitis C virus; IPH, Idiopathic portal hypertension; LC, liver cirrhosis; M, male; NA, not available; PH, portal hypertension; PT, prothrombin time; PVST, portal venous system thrombosis; SD, standard deviation; TBIL, total bilirubin.

Supplementary Table 4. Quality assessment of randomized studies using the Cochrane Collaboration's tool for assessing risk of bias.

Authors Journal (Year)		Ma J. Zhonghua Yi Xue Za Zhi (2008) Full-text	Wang HL. Can J Surg (2011) Full-text	Xue H. Zhonghua Wai Ke Za Zhi. (2000) Full-text
Selection bias.	Random sequence generation.	Unclear risk	Low risk	Unclear risk
	Allocation concealment.	Unclear risk	Low risk	Unclear risk
Performance bias.	Blinding of participants and personnel.	Unclear risk	High-risk	Unclear risk
Detection bias.	Blinding of outcome assessment.	Low-risk	Low-risk	Low-risk
Attrition bias.	Incomplete outcome data.	Unclear risk	High risk	Unclear risk
Reporting bias.	Selective reporting.	Unclear risk	Unclear risk	Unclear risk
Other bias.	Other sources of bias.	Unclear risk	Unclear risk	Unclear risk

Supplementary table 5. Quality assessment of non-randomized studies using Newcastle-Ottawa scale

Authors Journal (Year)		Kakinokil K. Surg Today (2013) Full-text	Kawanaka H. Ann Surg (2010) Full-text	Lai W. World J Gastroenterol. (2012) Full-text	Pan WD. J Gastroenterol Hepatol (2011) Abstract	Svensson M. Eur J Haematol (2006) Full-text
Selection	1) Representativeness of prevention group (the exposed cohort)	★	★	★	★	★
	2) Selection of no prevention group (the non exposed cohort)	★	★	★	★	★
	3) Ascertainment of patients receiving the prevention (exposure)		★			★
	4) Demonstration that PVST was not present at start of study	★	★			
Comparability	1) Comparability of cohorts on the basis of the design or analysis (sex and age as the most important fact, type of diseases as the second important factor)		★★	★★		
Outcome	1) Assessment of PVST outcome	★	★	★	★	★
	2) Was follow-up long enough for outcomes to occur (>1 month)		★			★
	3) Adequacy of follow up of cohorts (rate of follow up>80%)					
**Total score**		4 stars	8 stars	5 stars	3 stars	5 stars

Study or Subgroup	Prevention Events	Prevention Total	No prevention Events	No prevention Total	Weight	Odds Ratio M-H, Fixed, 95% CI
**1.2.1 Hematological diseases**						
Svensson M.	5	54	0	15	0.7%	3.44 [0.18, 65.86]
Wang HL.	1	15	0	14	0.5%	3.00 [0.11, 79.91]
**Subtotal (95% CI)**		**69**		**29**	**1.2%**	**3.27 [0.36, 29.57]**
Total events	6		0			
Heterogeneity: Chi² = 0.00, df = 1 (P = 0.95); I² = 0%						
Test for overall effect: Z = 1.05 (P = 0.29)						
**1.2.2 Portal hypertension**						
Kakinokil K.	10	14	12	14	3.4%	0.42 [0.06, 2.77]
Kawanaka H.	1	25	9	25	8.6%	0.07 [0.01, 0.64]
Lai W.	31	148	63	153	48.8%	0.38 [0.23, 0.63]
Ma J.	2	40	9	36	9.0%	0.16 [0.03, 0.79]
Pan WD.	10	56	19	56	15.6%	0.42 [0.18, 1.02]
Xue H.	0	36	13	35	13.5%	0.02 [0.00, 0.40]
**Subtotal (95% CI)**		**319**		**319**	**98.8%**	**0.29 [0.20, 0.43]**
Total events	54		125			
Heterogeneity: Chi² = 6.95, df = 5 (P = 0.22); I² = 28%						
Test for overall effect: Z = 6.19 (P < 0.00001)						
**Total (95% CI)**		**388**		**348**	**100.0%**	**0.33 [0.22, 0.47]**
Total events	60		125			
Heterogeneity: Chi² = 10.82, df = 7 (P = 0.15); I² = 35%						
Test for overall effect: Z = 5.89 (P < 0.00001)						
Test for subgroup differences: Chi² = 4.48, df = 1 (P = 0.03), I² = 77.7%						

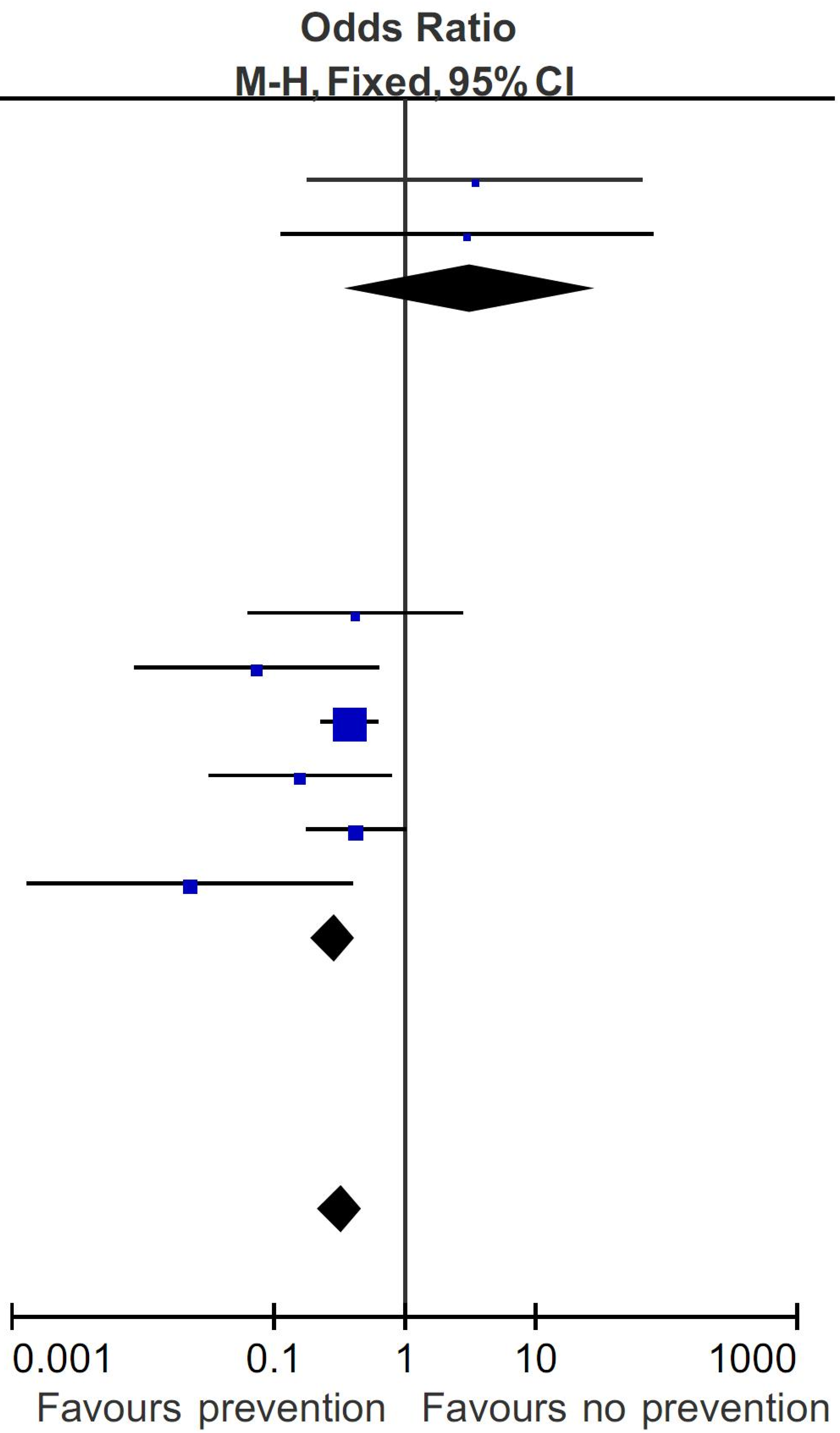

Study or Subgroup	Prevention Events	Prevention Total	Noprevention Events	Noprevention Total	Weight	Odds Ratio M-H, Fixed, 95% CI
**1.3.1 Anticoagulants**						
Kakinokil K.	10	14	12	14	3.4%	0.42 [0.06, 2.77]
Kawanaka H.	1	25	9	25	8.6%	0.07 [0.01, 0.64]
Lai W.	31	148	63	153	48.8%	0.38 [0.23, 0.63]
Pan WD.	10	56	19	56	15.6%	0.42 [0.18, 1.02]
Svensson M.	5	54	0	15	0.7%	3.44 [0.18, 65.86]
Wang HL.	1	15	0	14	0.5%	3.00 [0.11, 79.91]
**Subtotal (95% CI)**		**312**		**277**	**77.6%**	**0.40 [0.27, 0.59]**
Total events	58		103			
Heterogeneity: Chi² = 5.89, df = 5 (P = 0.32); I² = 15%						
Test for overall effect: Z = 4.53 (P < 0.00001)						
**1.3.2 Thrombolytics**						
Xue H.	0	36	13	35	13.5%	0.02 [0.00, 0.40]
**Subtotal (95% CI)**		**36**		**35**	**13.5%**	**0.02 [0.00, 0.40]**
Total events	0		13			
Heterogeneity: Not applicable						
Test for overall effect: Z = 2.58 (P = 0.010)						
**1.3.3 Prostaglandin E1**						
Ma J.	2	40	9	36	9.0%	0.16 [0.03, 0.79]
**Subtotal (95% CI)**		**40**		**36**	**9.0%**	**0.16 [0.03, 0.79]**
Total events	2		9			
Heterogeneity: Not applicable						
Test for overall effect: Z = 2.25 (P = 0.02)						
**Total (95% CI)**		**388**		**348**	**100.0%**	**0.33 [0.22, 0.47]**
Total events	60		125			
Heterogeneity: Chi² = 10.82, df = 7 (P = 0.15); I² = 35%						
Test for overall effect: Z = 5.89 (P < 0.00001)						
Test for subgroup differences: Chi² = 4.80, df = 2 (P = 0.09), I² = 58.4%						

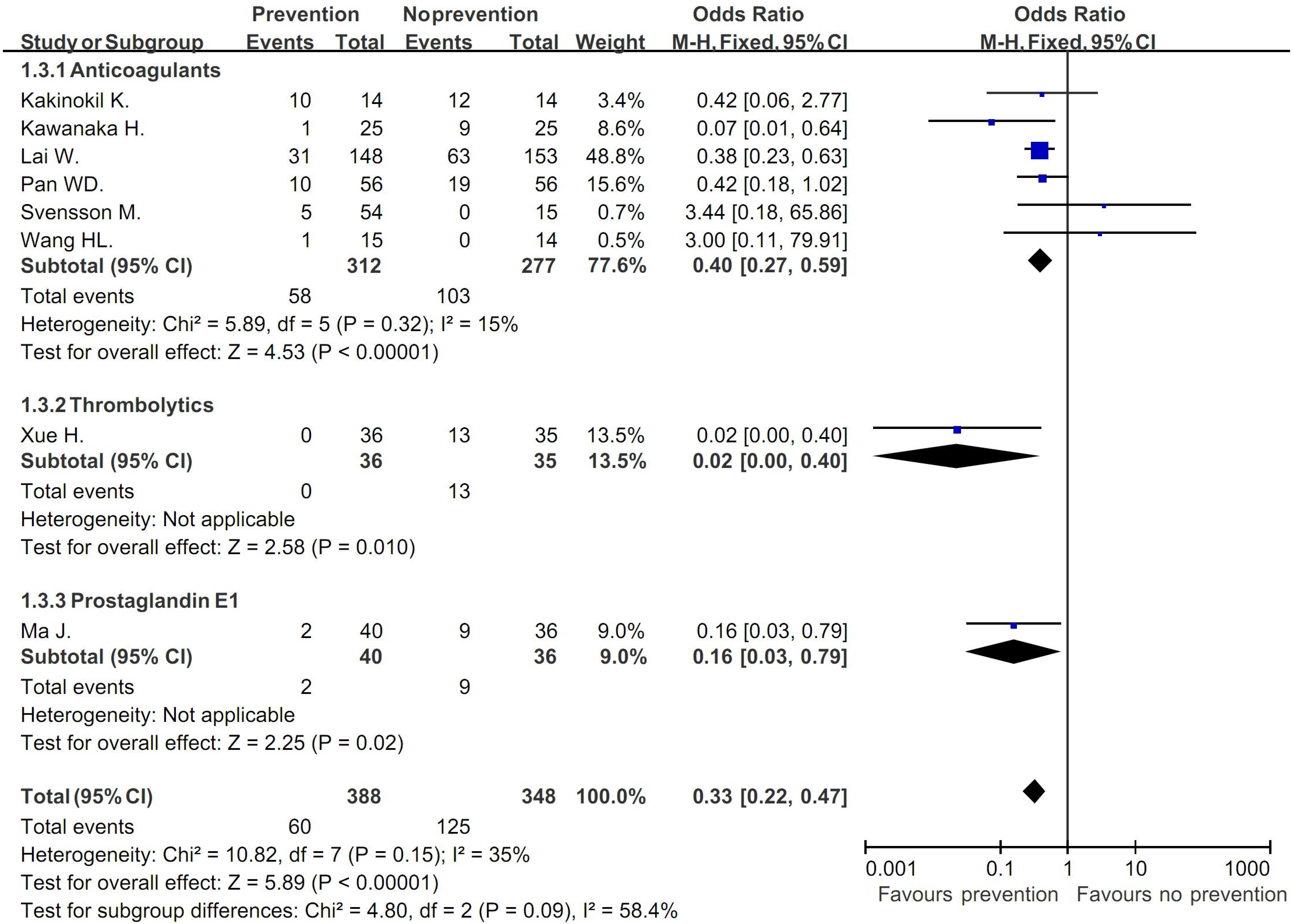

Study or Subgroup	Prevention Events	Prevention Total	Noprevention Events	Noprevention Total	Weight	Odds Ratio M-H, Random, 95% CI
**1.4.1 Randomized studies**						
Ma J.	2	40	9	36	12.0%	0.16 [0.03, 0.79]
Wang HL.	1	15	0	14	3.7%	3.00 [0.11, 79.91]
Xue H.	0	36	13	35	4.7%	0.02 [0.00, 0.40]
**Subtotal (95% CI)**		**91**		**85**	**20.4%**	**0.19 [0.02, 1.89]**
Total events	3		22			
Heterogeneity: Tau² = 2.43; Chi² = 4.93, df = 2 (P = 0.09); I² = 59%						
Test for overall effect: Z = 1.41 (P = 0.16)						
**1.4.2 Non-randomized studies**						
Kakinokil K.	10	14	12	14	9.5%	0.42 [0.06, 2.77]
Kawanaka H.	1	25	9	25	7.7%	0.07 [0.01, 0.64]
Lai W.	31	148	63	153	33.8%	0.38 [0.23, 0.63]
Pan WD.	10	56	19	56	24.2%	0.42 [0.18, 1.02]
Svensson M.	5	54	0	15	4.5%	3.44 [0.18, 65.86]
**Subtotal (95% CI)**		**297**		**263**	**79.6%**	**0.38 [0.24, 0.63]**
Total events	57		103			
Heterogeneity: Tau² = 0.04; Chi² = 4.41, df = 4 (P = 0.35); I² = 9%						
Test for overall effect: Z = 3.83 (P = 0.0001)						
**Total (95% CI)**		**388**		**348**	**100.0%**	**0.33 [0.17, 0.63]**
Total events	60		125			
Heterogeneity: Tau² = 0.27; Chi² = 10.82, df = 7 (P = 0.15); I² = 35%						
Test for overall effect: Z = 3.33 (P = 0.0009)						
Test for subgroup differences: Chi² = 0.34, df = 1 (P = 0.56), I² = 0%						

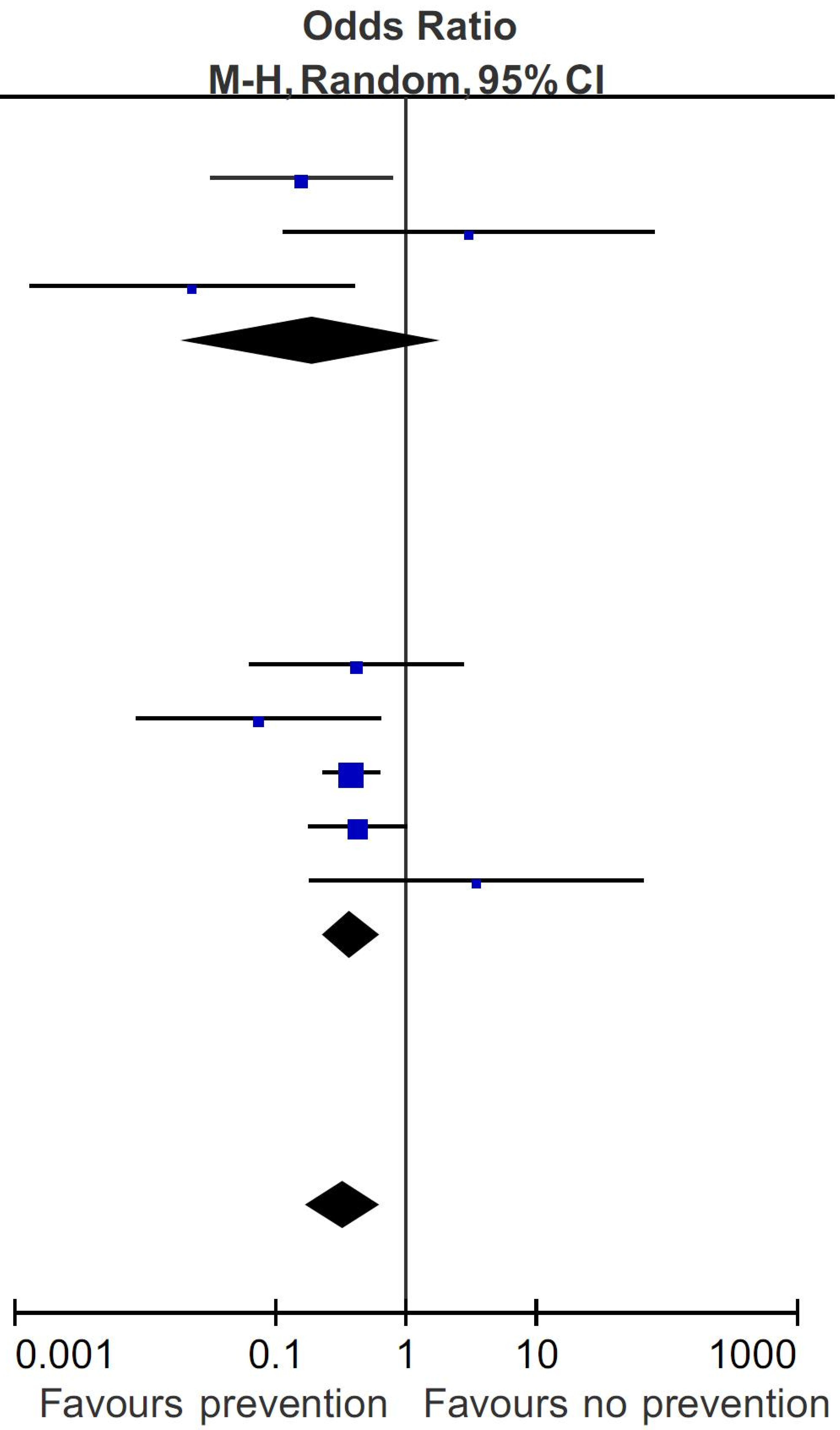

Supplement: Supplementary file 1 — Supplementary Figure 1: Forest plots showing the results of subgroup meta-analyses according to the type of study population comparing the incidence of portal venous system thrombosis after splenectomy between patients who received the preventive measures and those who did not. Supplementary Figure 2. Forest plots showing the results of subgroup meta-analyses according to the type of drugs comparing the incidence of portal venous system thrombosis after splenectomy between patients who received the preventive measures and those who did not. Supplementary Figure 3. Forest plots showing the results of subgroup meta-analyses according to the study design comparing the incidence of portal venous system thrombosis after splenectomy between patients who received the preventive measures and those who did not. [file 292689.f1.pdf]
